# Evaluating the multifaceted bioactivity of *Syzygium aromaticum* essential oil: the central role of eugenol

**DOI:** 10.55730/1300-0152.2728

**Published:** 2025-01-13

**Authors:** Noureddine RAHIM, Chamseddine DERABLI, Amina BRAMKI, Sara MAHDJOUB, Sandrine RUP-JACQUES, Ghozlane BARBOUCHA, Stephanie HESSE, Houssem BOULEBD

**Affiliations:** 1 Higher National School of Biotechnology Taoufik Khaznadar, New University Pole Ali Mendjeli, Biotechnology Laboratory, Constantine, Algeria; 2 Higher National School of Biotechnology Taoufik Khaznadar, New University Pole Ali Mendjeli, Laboratory of Bioengineering, Constantine, Algeria; 3 LCP-A2MC, University of Lorraine, Metz, France; 4 Department of Chemistry, Faculty of Exact Sciences, University of Frères Mentouri Constantine 1, Constantine, Algeria

**Keywords:** *Syzygium aromaticum*, eugenol, biological activity, DFT calculations

## Abstract

**Background/aim:**

*Syzygium aromaticum* L. is a versatile plant traditionally used to treat digestive and respiratory issues, improve oral health, and relieve pain, particularly in regions such as Southeast Asia, South Asia, and parts of the Middle East. Its essential oil (EO), predominantly composed of eugenol, is rich in bioactive compounds. This study aims to clarify the specific contribution of eugenol to the antioxidant, antibacterial, antifungal, and insecticidal activities of *S. aromaticum* EO by comparing their individual effects. Additionally, density functional theory (DFT) calculations were employed to examine the antioxidant mechanism of eugenol and its role in enhancing the overall activity of the EO.

**Materials and methods:**

The EO was obtained from *S. aromaticum* and analyzed using GC-MS to determine its composition. Antioxidant activity was assessed through the DPPH scavenging assay. Antibacterial and antifungal activities were evaluated using the disk diffusion method against various strains, while insecticidal and repellent effects were tested on *Bruchus lentis* at different concentrations and exposure times. Antioxidant mechanisms were investigated using DFT calculations

**Results:**

The findings underscore the strong antioxidant, antibacterial, antifungal, and insecticidal properties of *S. aromaticum* EO, with eugenol identified as the primary active component driving the antioxidant and insecticidal effects. Additionally, eugenol has been found to exhibit moderate scavenging activity in lipid media. However, its activity is higher in polar media, with a *k*_overall_ = 1.70 ⊠ 10^6^ M^−1^s^−1^ comparable to that of ascorbic acid. The single-electron transfer mechanism from the deprotonated state was found to play a decisive role under these conditions.

**Conclusion:**

*S. aromaticum* EO exhibits remarkable antioxidant, antibacterial, antifungal, and insecticidal activities. A significant portion of these properties can be attributed to the presence of eugenol. This suggests that eugenol plays a critical role in the EO’s overall efficacy, making *S. aromaticum* a promising candidate for applications in natural health products, pharmaceuticals, and agricultural pest management.

## 1. Introduction

Since ancient times, humans have been captivated by natural compounds, particularly, essential oils (EOs), which remain of paramount importance today (de Sousa et al., 2023). EOs are aromatic, volatile substances isolated from various parts of plants (Hyldgaard et al., 2012). Their diverse chemical compositions and structures allow them to be utilized in multiple domains, including agriculture, food preservation, cosmetics, and pharmaceuticals (Bakkali et al., 2008; Ríos, 2016; Yadav et al., 2017). For example, in the food industry, EOs offer a natural alternative to synthetic additives, enhancing both shelf life and flavor. Additionally, their antimicrobial and antioxidant properties make them useful in food packaging materials to prevent spoilage (Saeed et al., 2022). Pharmaceutically, EOs are recognized for their therapeutic potential in treating various conditions, such as rheumatism, ulcers, hypertension, and hyperemia (Pauli and Schilcher, 2004; Bonamin et al., 2014; Barão Paixão and Freire de Carvalho, 2021). In agriculture, EOs are used as an alternative to conventional synthetic chemical pesticides due to their broad spectrum of activity against pest insects and plant pathogenic fungi, with the added benefits of biodegradability and lower health risks (Moharramipour and Negahban, 2014; Benelli et al., 2018). In light of their considerable value, EOs continue to attract interest from researchers across diverse fields.

Among the various EO ranges, the genus Syzygium, comprising approximately 1,800 species primarily found in the tropical regions of Asia (Kadir et al., 2022), holds significant economic and medicinal value due to its diverse phytochemical composition and remarkable biological properties (da Costa et al., 2020; Kadir et al., 2022). Historically, species from this genus have been used in Indian folk medicine to treat ailments such as cough, diabetes, dysentery, and inflammation (Ayyanar and Subash-Babu, 2012). One of the most notable species is *S. aromaticum*, commonly known as clove, native to the Maluku Islands in Indonesia. Today, it is cultivated in several parts of the world including India, Malaysia, Sri Lanka, Madagascar, and Tanzania (Cortés-Rojas et al., 2014). The dried flower buds are widely used as a spice, while clove EO has been traditionally applied to treat burns and alleviate pain in dental care, particularly in treating tooth infections and toothaches. Moreover, clove EO is utilized across various industries, including the production of perfumes, soaps, and cleaning agents, as well as in histological procedures (Batiha et al., 2020). Its various biological applications, particularly its antimicrobial and insecticidal properties, are primarily attributed to its high eugenol content (Machado et al., 2011; Rana et al., 2011). Eugenol, the major bioactive compound in clove EO, is widely applied in the food industry as a flavoring agent, in medicine for its antiinflammatory and analgesic properties, and in agriculture as an antiparasitic, acaricidal, and insecticidal agent (Huang et al., 2002; Maurya et al., 2018; Shang et al., 2021).

In the last five years, several studies have highlighted the diverse phytochemical composition and biological activities of *S. aromaticum* EO. Beyond eugenol, which typically constitutes the major component, clove EO contains other bioactive compounds such as β-caryophyllene, humulene, and methyl salicylate, among others (Yadav et al., 2022; Ullah et al., 2023). These constituents contribute to its multifaceted biological properties. Several pharmacological properties of *S. aromaticum* have been reported, including antiseptic, antimutagenic, antiinflammatory, antioxidant, antiulcerogenic, antithrombotic, antifungal, antiviral, and antiparasitic activities. Recent research has demonstrated its potent antimicrobial activity against multidrug-resistant bacterial strains and pathogenic fungi, often surpassing the efficacy of standard treatments (Shahina et al., 2022; Cruz et al., 2024). Clove EO has also displayed cytotoxic effects against cancer cell lines, as well as antimutagenic activities, which further emphasize its therapeutic potential (Abadi et al., 2022). Additionally, studies have explored its antiinflammatory effects, with findings suggesting its potential in reducing cytokine production and inflammation-related enzymes (Elbestawy et al., 2023).

In this context, the present study aims to evaluate the antioxidant, antifungal, antibacterial, insecticidal, and repellent activities of *S. aromaticum* EO and its main compound, eugenol, against *Bruchus lentis* Frölich (Coleoptera: Bruchidae), a significant insect pest of stored lentils (*Lens culinaris* L.). To the best of our knowledge, this is the first investigation into the insecticidal potential of *S. aromaticum* EO and eugenol against this storage pest. In addition, we present a detailed analysis of the antioxidant mechanism of eugenol, particularly focusing on how different physiological conditions influence its antioxidant activity. This study also provides a novel comparison between the essential oil and its primary component, eugenol, offering valuable insights into the distinctive biological properties of these compounds. Our research aims to fill a gap in the current literature by comprehensively assessing these activities and providing a better understanding of the modes of action involved.

## 2. Materials and methods

### 2.1. Extraction of essential oil

*S. aromaticum* buds were purchased from a local market in Constantine (North Eastern Algeria). The buds were subjected to quality control, including visual inspection, olfactory evaluation, and moisture content measurement to ensure freshness and stability. Only high-quality, intact *S. aromaticum* buds were cleaned, sorted, homogenized, and ground into a fine powder using a laboratory mill to optimize EO extraction.

The EO was obtained through hydrodistillation of 50 g of powdered material using a Clevenger-type apparatus at 100 °C for 3 h (Suttiarporn et al., 2024). The distillate was then extracted with CH_2_Cl_2_, dried over anhydrous Na_2_SO_4_, and the CH_2_Cl_2_ was then evaporated under reduced pressure. The *S. aromaticum* EO was obtained and refrigerated at 4 °C until analysis. The resulting oil was stored in a refrigerator at 4 °C in an amber-colored glass bottle until it was ready for use in GC–MS characterization and lab experiments (Saad et al., 2022). The EO yield was determined as the ratio of the weight of oil to the weight of *S. aromaticum* buds.


$$\text{Yield}(\%)=\frac{\text{weight of collected extract}}{\text{dry weight of the extracted sample}}\times 100$$

Eugenol, with a purity more than 98%, used in this study, was obtained from Sigma-Aldrich (Hamburg, Germany).

### 2.2. Gas chromatography-mass spectrometry (GC-MS) analysis

The separation and identification of the EO constituents were conducted using gas chromatography coupled with mass spectrometry (GC-MS) on a Clarus 500 GC/MS, equipped with a single Elite 5MS capillary column (30 m length, 0.25 mm internal diameter, 0.25 μm film thickness). The injector temperature (Split 1/20) was set to 250 °C, and the MS source temperature to 250 °C. Helium was used as the carrier gas at a flow rate of 0.75 mL/min, with an injection volume of 0.1 μL for the EO diluted at 1/200 in ether. The injection mode was Split (1/60).

The column temperature was programmed from 60 °C for 2.5 min then increased to 250 °C at a rate of 10 °C/min and then held at 300 °C for 5 min. Electron impact ionization at 70 eV was used for fragmentation. Identification was based on comparing the mass spectra obtained for each EO compound with a reference spectrum database.

### 2.3. Antioxidant assay

The test was carried out with a radical of DPPH. The DPPH assay was performed following the method previously described by Blois (1958). Test sample (1 mg of *S. aromaticum* EO and eugenol) was dissolved in 1 mL of methanol. The reaction mixture contained 160 μL of 0.1 mM DPPH and 40 μL of the sample solution (*S. aromaticum* EO and eugenol) in methanol. After a 30-min incubation at room temperature, the absorbance of the resulting mixture was measured at 515 nm using a FLUOstar Omega multimode fluorescence microplate reader (BMG Labtech, Germany). Control samples, consisting of 40 μL of methanol in DPPH solution, were measured at the same wavelength. The percentage of inhibition was calculated using the following equation:


$$\text{I}=\left[\frac{{\text{A}}_{\text{B}}-{\text{A}}_{\text{A}}}{{\text{A}}_{\text{B}}}\right]\times 100$$

I: Percentage of DPPH inhibition; A_B_: Absorbance of the blank (at t = 0 min); A_A_: Absorbance of the tested extract (at t = 20 min).

The IC_50_ value, indicating concentration dependence, was determined for the EO and its derivative, eugenol, using the DPPH radical to achieve 50% inhibition compared to the control. Ascorbic acid and Trolox served as reference standards.

### 2.4. Quantum chemistry calculations

All density functional theory (DFT) calculations in this study were conducted using Gaussian 09 software (Frisch et al., 2009). The M06-2X functional, paired with the 6-311++G(d,p) basis set, was employed for all radical scavenging mechanism calculations (Zhao and Truhlar, 2008b). Previous studies have validated the robustness of this approach, particularly in reactions involving free radicals (Zhao and Truhlar, 2008a; Galano and Alvarez-Idaboy, 2014). The identification of ground and transition states was confirmed by analyzing imaginary frequencies. To simulate both polar and lipid physiological conditions, solvation effects of water and pentyl ethanoate were integrated using the SMD solvation model (Marenich et al., 2009). Thermodynamic descriptors related to the studied antioxidant mechanisms were calculated in accordance with established methodologies (Boulebd 2022a; 2022b). Kinetic evaluations were carried out following the quantum mechanics-based overall free radical scavenging activity (QM-ORSA) approach (Alberto et al., 2013; Galano and Alvarez-Idaboy, 2013). The rate constant (*k*) was determined using standard transition state theory at a 1 M standard state and 298.15 K, calculated according to the equation below (Evans and Polanyi, 1935; Eyring, 1935; Truhlar et al., 1983):


$$k=\sigma \kappa \frac{k_{B}T}{h}e^{-\frac{\Delta G^{\neq }}{RT}},$$

where σ represents the reaction symmetry number, κ accounts for tunneling corrections computed using the Eckart barrier (Eckart, 1930), k_B_ is the Boltzmann constant, h is the Planck constant, and ΔG^‡^ is the Gibbs free energy of activation.

### 2.5. Antimicrobial activity

The antimicrobial activity of *S. aromaticum* EO and its primary component, eugenol, was evaluated using the disk diffusion method against two bacterial strains, *Bacillus cereus* (ATCC 11778) and *Staphylococcus aureus* (ATCC 25923), as well as two fungal isolates, *Aspergillus niger* (MH109542) and *Trichoderma* sp. Bacterial suspensions were prepared from 18-h cultures. The cell density of suspensions was adjusted by diluting in sterile physiological saline, and compared to the 0.5 McFarland standard (Bramki et al., 2019). Conversely, the fungal suspensions were obtained from 14-day cultures by scraping the colonies after addition of 10 mL of physiological saline. The suspensions were then diluted until the absorbance reached 0.15 to 0.2 at 650 nm. Subsequently, these suspensions were further diluted to 1/10th (Barboucha et al., 2024). The test was performed on Petri dishes containing Muller-Hinton previously seeded with the bacterial strains, and Sabouraud medium seeded with fungal isolates. Afterward, 6 mm diameter discs of Whatman paper were soaked with 20 μL of each product. The plates were kept in the fridge for 2 h, and then incubated at 37 °C for 24 h for the bacterial strains, while the molds were incubated at 28 °C for 48 to 72 h. The antimicrobial activity was determined by measuring the inhibition zone surrounding the disks in millimeters (Sehout et al., 2021).

### 2.6. Insecticidal activity

#### 2.6.1. Test insect culture

The lentil weevil *Bruchus lentis* Frölich (Coleoptera: Bruchidae) was selected for this study because it is one of the most important pests of lentil, causing severe damage worldwide (Saeidi and Mirfakhraie, 2017). Appearing in high populations in fields and storage facilities, *B. lentis* can cause economic losses of up to 40% of the lentil crop (Gorham, 1991). Its persistence in stored lentils is attributed to its rapid reproductive rate, adaptability to various storage environments, and resistance to conventional chemical control measures (Aidbhavi et al., 2023). Given its economic impact, comprehensive research on *B. lentis* is essential for developing effective and sustainable pest management strategies. Such approaches are crucial for preserving lentil quality, reducing postharvest losses, and minimizing reliance on chemical pesticides.

*B. lentis* used in this study was obtained from starter colonies at the wholesale grain market in Constantine, Algeria. Lentil grains infested with the weevil were placed in glass jars (20 cm × 8 cm) with perforated lids to ensure proper ventilation and stored under room conditions of 27± 2 °C, 70 ± 5% relative humidity, and L:D = 12:12 h. For toxicity testing, newly emerged adults of both sexes (<72 h old) were used. All tests were carried out under the same environmental conditions as the cultures. In all bioassays, insects were considered dead when no leg or antennal movements were observed.

#### 2.6.2. Contact toxicity

The tests were conducted following the protocol described by Zhang et al. (2021). In order to determine the appropriate doses of *S. aromaticum* EO and its main constituent, eugenol, preliminary tests were carried out. Four concentrations were prepared by dissolving 10, 20, 40, and 60 μL of EO or eugenol in 1 mL of acetone. A volume of 1 μL from each concentration was carefully applied to the insect’s thorax using a micropipette to ensure precise dosing. Acetone served as the control. Each treatment and control group consisted of ten insects, and the experiment was conducted in triplicate. Following application, both treated and control insects were placed in Petri dishes (90 mm in diameter) containing 40 g of sterilized crushed lentil seeds. To prevent fumigant toxicity, the Petri dishes were sealed with perforated plastic. The contact toxicity concentration was defined as the volume of EO (in microliters) necessary to induce a toxic effect per adult insect. Insect mortality percentages were calculated using Abbott’s correction formula to account for natural mortality in untreated controls (Abbott, 1925). Additionally, lethal concentrations (LC50 and LC90) and their corresponding 95% confidence intervals were determined.

Corrected mortality (%) was calculated using Abbott’s formula:


$$\text{Corrected mortality }(\%)=(1-\frac{\text{n in T after treatment}}{\text{n in Co after treatment}})\times 100,$$

where *n* represents the number of insects, *T* denotes the treated group, and *Co* refers to the control group.

#### 2.6.3. Repellent activity

The repellent activity of *S. aromaticum* EO and its major compound, eugenol, against *B. lentis* adults was assessed using the method described by McDonald et al. (1970). For both treatments, EO and eugenol were diluted in 500 μL of acetone at concentrations of 2, 4, 6, and 8 μL, corresponding to 0.003, 0.006, 0.013, and 0.02 mg/cm^2^, respectively. A 9 cm diameter Whatman paper disc (No. 1) was divided into two equal halves, with one half treated with 500 μL of the prepared sample and the other half receiving an equal volume of acetone as a control. After a 10 min drying period, the two halves were reassembled and placed inside Petri dishes. Ten adult insects were introduced at the center of each disc, and their distribution on the treated and control halves was recorded 2 h posttreatment. Each experiment was performed in triplicate.

The repulsion percentage (RP) was calculated using the formula described by McDonald et al. (1970):


$$\text{RP}=\frac{\left(\text{NC}-\text{NT}\right)}{\left(\text{NC}+\text{NT}\right)}\times 100,$$

where PR represents the percentage of repellence, NC is the number of test insects on the control half disc, and NT is the number of insects on the treated half of the disc.

### 2.7. Formulation of herbal cream

Due to the powerful antioxidant, antibacterial, and antifungal properties of *S. aromaticum* EO, an herbal cream containing this EO has been formulated.

#### 2.7.1. Preparation

The oil-in-water type cream was prepared according to the formula presented in Table 1. The oil phase and the aqueous phase were prepared in two separate beakers and then brought to the same temperature in a water bath heated to 60–70 °C for about 10 min, stirring occasionally. Adding the preservative to the aqueous phase was considered an optimal choice to prevent the proliferation of bacteria and molds. Once both phases reached the same temperature, the aqueous phase was poured into the oil phase while stirring vigorously. After removing the mixture from the water bath, the mixture was then stirred using an electric mixer in an ice bath until it cooled to room temperature and formed the cream. In this step, vitamin E was added to prevent rancidity caused by free radicals in the oils. While continuing to stir to thoroughly incorporate the ingredients, clove essential oil was added last, before the final product was transferred and stored in a sterile glass jar.

#### 2.7.2. Quality control

The main objective of quality control is to detect, evaluate, and correct errors due to flaws in the analysis system or environmental conditions during manufacturing. The formulated cream was evaluated through various analyses and parameters such as organoleptic, microscopic, and microbiological analysis, pH, and centrifugation stability (see supplementary materials) (Khan et al., 2013; Matangi et al., 2014; Colucci et al., 2020).

### 2.8. Statistical analysis

All experiments were conducted in triplicate. The results were analyzed using one-way analysis of variance (ANOVA), followed by Tukey’s HSD post hoc test for multiple comparisons, with the normality of data distribution and homogeneity of variances verified prior to analysis. Differences were considered significant at p < 0.05. Insect mortality data recorded were further subjected to probit analysis to calculate the LC_50_ and LC_90_ values. All statistical analyses were conducted using SPSS software version 25.0.

## 3. Results and discussion

### 3.1. Chemical composition of *S. aromaticum* essential oil

The *S. aromaticum* EO was yellow, with a yield of 8.9% (0.18 mL/g). Our results align with the standards of the European Pharmacopoeia, which require a minimum content of 0.15 mL/g (7.4%) of dry material. The yield obtained is slightly lower than that reported by González-Rivera et al. (2016), who achieved a yield of 11%. The yield and quality of the oil can be influenced by various factors, including the origin, variety, and quality of raw materials, the distillation method used, and post distillation processing (Peter, 2012).

**Figure 1:**
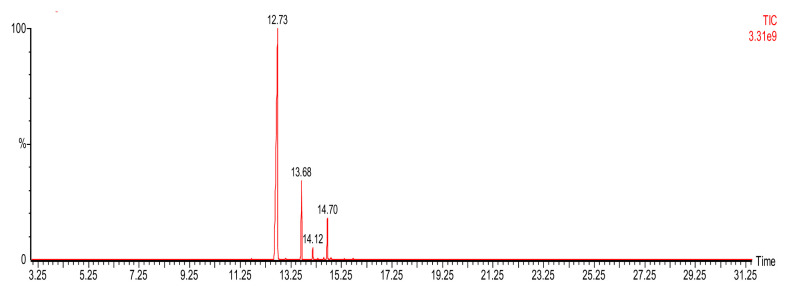
Chromatogram of GC-MS of S. *aromaticum* EO.

The gas chromatography-mass spectrometry (GC-MS) analysis enabled a quantitative assessment of the EO composition (Figure 1). This analysis revealed a total of 18 chemical compounds (Figure 1) with a dominance of two constituents: eugenol (79.21%) and β-caryophyllene (11.60%), followed by eugenol acetate (6.27 %), and humulene (1.56%) (Table 2)

Several studies have reported that the main constituents of *S. aromaticum* EO are eugenol, β-caryophyllene, and eugenol acetate, with varying concentrations. Hossain et al. (2012) analyzed the chemical composition of clove EO extracted by hydrodistillation from two samples sourced from India and Indonesia. They identified the primary constituents as eugenol (51.51% in the Indian sample and 46.53% in the Indonesian sample) and β-caryophyllene (36.20% and 43.03%, respectively). Similarly, Xie et al. (2015) identified eugenol (90.6%) and β-caryophyllene (9.4%) as the predominant constituents in *S. aromaticum* EO from China. Ahamad (2023) found that the major components of *S. aromaticum* EO from Iraq were eugenol (59.87%), β-caryophyllene (23.58%), α-selinene (4.67%), α-terpinyl acetate (4.12%), and humulene (3.74%). In a study of clove EO from Algeria, Selles et al. (2020) identified eugenol (78.72%), β-caryophyllene (8.82%), and eugenol acetate (8.74%) as the primary constituents. Additionally, Uchôa Lopes et al. (2020) analyzed *S. aromaticum* EO from Brazil and identified eugenol as the major compound, accounting for 84.63%, followed by eugenol acetate at 11.37%.

This variability in the chemical composition of EO can be attributed to factors such as the maturity stage of the plant, genetic variability, environmental conditions (e.g., temperature, photoperiod, and hygrometry), analytical methods, and the significant influence of geographical and ecological variations across habitats.

### 3.2. Antioxidant activity

**Figure 2:**
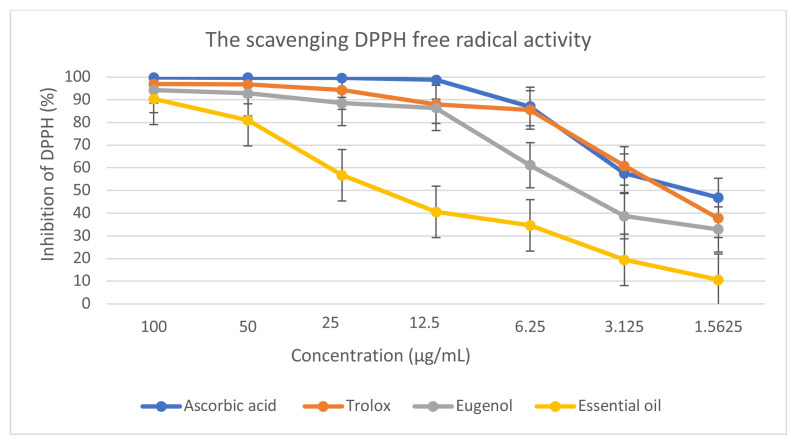
Inhibition of the DPPH radical by *S. aromaticum* EO and eugenol.

*S. aromaticum* EO and eugenol presented concentration-dependent antioxidant activity, as shown in the graph that relates *S. aromaticum* EO and eugenol concentration to the percentage of inhibition of the DPPH radical (Figure 2). The findings showed that *S. aromaticum* EO exhibits notable free radical scavenging activity with an IC_50_ of 15.07 ± 2.12 μg/mL. Notably, eugenol, the primary component of the oil, exhibited an IC_50_ of 5.60 ± 1.91 μg/mL, demonstrating a radical scavenging capacity nearly three times stronger than the EO, thus highlighting its significant role in enhancing the oil’s antioxidant properties. Although eugenol’s activity is comparable to standard antioxidants, ascorbic acid and Trolox, with IC_50_ values of 2.04 ± 0.26 μg/mL and 3.43 ± 0.38 μg/mL, respectively. These standards still show superior antioxidant capacities compared to *S. aromaticum* EO.

Eugenol and *S. aromaticum* EO exhibited a strong ability to eliminate DPPH radicals, consistent with previous findings on the antioxidant activity of clove EOs that are rich in eugenol (Teles et al., 2021; Kiki, 2023). Similar results were reported in a study assessing the antioxidant potential of EOs derived from different parts of the *S. aromaticum* plant, which demonstrated their effectiveness in reducing DPPH radicals (Alfikri et al., 2020). Our study corroborates the prominent role of eugenol in the antioxidant profile of *S. aromaticum* EO, aligning with other researches that identifies eugenol as the primary bioactive compound in clove (Kaur et al., 2019; El Ghallab et al., 2020).

### 3.3. Evaluation of the antioxidant mechanism

**Figure 3:**
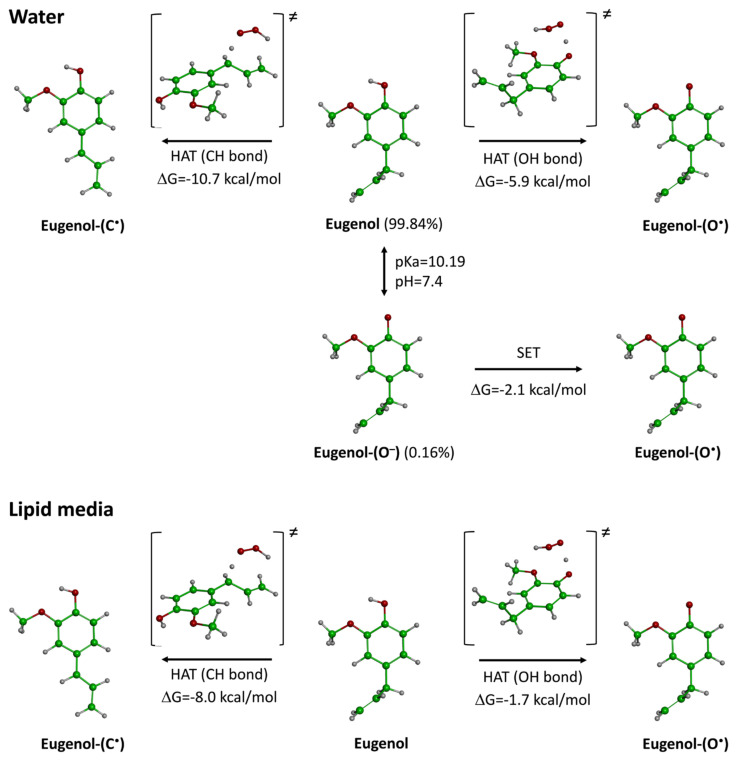
Thermodynamic behavior of the radical scavenging mechanisms of eugenol in physiological media.

To explain the potent antioxidant activity of eugenol, the primary compound in *S. aromaticum* EO, kinetic calculations based on density functional theory (DFT) were performed using the QM-ORSA protocol (Alberto et al., 2013; Galano and Alvarez-Idaboy, 2013). Although some studies have explored its antioxidant mechanism, some critical aspects remain unclear, including the influence of physiological conditions, the role of acid-base equilibrium, and the kinetics of hydrogen atom transfer (HAT) for the benzylic C–H bond (Boulebd, 2019). The results of our calculations are presented in Figure 3, while the kinetic data are summarized in Table 3.

Eugenol has two potential sites for the HAT mechanism: the hydroxyl (O–H) bond and the benzylic C–H bond. In a previous study, we demonstrated that the C–H bond is the thermodynamically preferred site for free radical attack (Boulebd, 2019). However, recent studies (Boulebd et al., 2021; Boulebd, 2023) have highlighted the faster kinetics of O–H bonds compared to C–H bonds, prompting us to include both sites in this kinetic study. The acid-base equilibrium of eugenol may also influence its antioxidant activity, especially in polar physiological environments. The reported experimental pKa of eugenol (10.19) (Kortüm et al., 1960) indicates that it exists primarily in its neutral form at physiological pH (99.84%), although a small fraction (0.16%) may exist in its deprotonated form. Therefore, this deprotonated state was also considered in polar physiological media. As shown in Figure 3, the HAT mechanism is dominant in both polar and lipid environments, with Gibbs free energy (ΔG) values ranging from −1.7 to −10.7 kcal/mol. The C–H bond is thermodynamically more favorable in both media (−8.0 to −10.7 kcal/mol vs. −1.7 to −5.9 kcal/mol), consistent with our previous findings (Boulebd et al., 2021). The single-electron transfer (SET) mechanism from the deprotonated state was also found to be favorable, with a ΔG of −2.2 kcal/mol (Spiegel, 2022).

According to Table 3, the overall rate constant (*k*_overall_) of eugenol in lipid media is 9.00 × 10^3^ M^−1^ s^−1^, which is lower than that of BHT (1.70 × 10^4^ M^−1^ s^−1^) (Boulebd, 2022b) and feruloylquinic acid (4.10 × 10^4^ M^−1^ s^−1^) (Boulebd et al., 2023). This suggests that eugenol acts as a moderate antioxidant in lipid environments.

In water, the SET mechanism appears to play a significant role in radical scavenging with a branching ratio of nearly 100%, indicating that SET is the exclusive mechanism in polar media. The overall rate constant *k*_overall_ of eugenol in water is 1.16 × 10^7^ M^−1^ s^−1^, comparable to that of ascorbic acid (9.97 × 10^7^ M^−1^ s^−1^) (Galano and Alvarez-Idaboy, 2013), and higher than that of Trolox (1.30 × 10^5^ M^−1^ s^−1^) (Alberto et al., 2013), BHT (1.51 × 10^5^ M^−1^ s^−1^) (Boulebd, 2022b), and cannabidiolic acid (2.40 × 10^6^ M^−1^ s^−1^) (Boulebd, 2022a). These results highlight eugenol’s strong antioxidant activity, consistent with experimental studies. Based on these findings, we conclude that eugenol is a potent antioxidant in polar physiological media, acting predominantly through the SET mechanism.

### 3.4. Antimicrobial activity

The antimicrobial activity test, carried out using the disk diffusion method, revealed the sensitivity of the four tested strains to clove EO and its main compound, eugenol. The results, presented as diameters of inhibition zones (in millimeters), are shown in Table 4.

The results showed that, for the bacterial strain *B. cereus*, the inhibition zone for *S. aromaticum* EO was 27.7 mm, compared to 24.6 mm for eugenol, with a statistically significant difference (one-way ANOVA, F = 40.5, p = 0.03), indicating the superior efficacy of the EO. Similarly, against *S. aureus*, the EO exhibited a significantly larger inhibition zone of 31.3 mm, while eugenol produced 21.8 mm (one-way ANOVA, F = 649.8, p < 0.005), highlighting the EO’s substantially stronger antibacterial activity (Table 4).

Regarding antifungal activity, EO showed an inhibition zone of 23.5 mm against *A. niger* compared to 19.3 mm for eugenol (F = one-way ANOVA, 89.3, p = 0.01), confirming its enhanced antifungal properties. Against *Trichoderma* sp., the EO exhibited an inhibition zone of 23.2 mm, while eugenol produced 18.5 mm (one-way ANOVA, F = 196.0, p < 0.005), further demonstrating the EO’s significantly greater antifungal activity.

According to our findings, *S. aromaticum* EO consistently demonstrates greater antibacterial and antifungal activity than eugenol alone. This could be due to the synergistic effect of the different compounds present in the EO, making its antimicrobial action more powerful compared to that of isolated eugenol. Similar synergistic interactions have been reported in previous studies. For example, Sharma et al. (2020) highlighted the enhanced antimicrobial efficacy of EOs due to the complex interplay of their components, which can act additively or synergistically to disrupt microbial membranes. Marchese et al. (2017) observed that the antimicrobial activity of clove EO was not solely attributed to eugenol but also to the contribution of other phenolic compounds and terpenes, which together enhanced its efficacy. Tyagi and Malik (2011) reported that main active compounds in EO are known to be responsible for the antimicrobial activity, while minor compounds may also contribute through synergistic or additive effects. Furthermore, Maggini et al. (2024) reported that *S. aromaticum* EO contains a variety of active compounds, eugenol being the principal component; however, other synergistic molecules also contribute to the biological properties of the EO. In fact, *S. aromaticum* EO and eugenol are well-known for their antimicrobial activity, which was proven by several researchers including Bai et al. (2023), who demonstrated that both *S. aromaticum* EO and eugenol exhibited a potent inhibitory effect against *Escherichia coli* and *S. aureus*. Moreover, Haro-González et al. (2021) demonstrated that *S. aromaticum* EO can exhibit a strong antimicrobial activity against several microorganisms, including both gram-negative and gram-positive bacteria. Additionally, it is effective against yeasts and molds due to its lipophilic properties. EOs can disrupt membrane structures, leading to a loss of stability and leakage of cellular contents, ultimately resulting in cell death (Morsy, 2017).

### 3.5. Insecticidal activity

#### 3.5.1. Contact toxicity

The effect of *S. aromaticum* EO and its major compound, eugenol, on the mortality of *B. lentis* was evaluated across various doses (μL/insect) and exposure times (24 h, 48 h, and 72 h) (Table 5). The data showed that the mortality results of *B. lentis* following the application of *S. aromaticum* EO and eugenol revealed notable differences between the two treatments, particularly in relation to the applied doses and exposure time. At the lowest dose (0.01 μL/insect), the EO demonstrated moderate mortality after 24 h (10%), gradually increasing to 21.7% at 72 h. In contrast, eugenol at the same dose resulted in a higher initial mortality rate (36.67% at 24 h), reaching 60% by 72 h, indicating a greater efficacy at lower concentrations.

At higher doses (0.04 and 0.06 μL/insect), both treatments exhibited high mortality rates. The EO achieved 100% mortality after 72 h at 0.06 μL/insect, similar to eugenol, which reached 100% as early as 48 h at this dose. However, eugenol appeared to induce faster mortality overall, particularly at the 0.06 μL/insect dose. The Tukey tests confirm that the differences are statistically significant, especially at higher doses, where different letters indicate variations between the two treatments and across exposure times.

Probit analysis revealed that at 72 h, EO had an LC50 of 0.021 μL/insect and an LC90 of 0.049 μL/insect, while eugenol exhibited greater potency with an LC50 of 0.008 μL/insect and an LC90 of 0.041 μL/insect (Table 6). These results suggest that eugenol is more effective than EO, requiring lower concentrations to achieve similar mortality rates.

The effect of *S. aromaticum* EO and eugenol, on the mortality of *B. lentis* aligns with findings from other studies that have demonstrated the insecticidal potential of *S. aromaticum* EO and eugenol. In this study, eugenol consistently outperformed EO, showing higher efficacy at lower concentrations and faster mortality rates. These results are supported by previous research, which has reported that eugenol is a potent bioactive compound with strong insecticidal properties due to its ability to disrupt insect neurological functions and affect respiratory systems (Alimi et al., 2023). Huang et al. (2002) revealed the effectiveness of clove EO against stored-product insects, highlighting eugenol’s role as the primary insecticidal component. Similarly, Rodríguez et al. (2022) emphasized that EOs rich in phenolic compounds like eugenol exhibit significant insecticidal activity. This supports the findings of this study, where eugenol achieved 100% mortality of *B. lentis* at a lower dose and in a shorter exposure time compared to EO. The observed LC50 and LC90 values are consistent with previous literature, indicating that eugenol is more potent than the whole EO. Sousa et al. (2022) also found that eugenol was more effective against various insect species, including *Trioza erytreae* (Hemiptera: Triozidae). The higher efficacy of eugenol can be attributed to its smaller molecular size, which may allow it to penetrate insect cuticles more easily, leading to faster mortality (Reynoso et al., 2023).

#### 3.5.2. Repellency activity

The repellent efficacy of *S. aromaticum* EO and its primary compound, eugenol, against *B. lentis* adults is presented in Table 7.

The data showed that both *S. aromaticum* EO and eugenol demonstrated a dose-dependent repellent effect on *B. lentis*, with increasing doses corresponding to higher repulsion percentages observed 2 h postapplication.

At the lowest concentration (1%), *S. aromaticum* EO and its main compound, eugenol, showed moderate repellent activity, with repulsion rates of 25% and 26.7%, respectively. However, at a concentration of 2%, the repellent effect became more pronounced, with eugenol (51.67%) slightly outperforming *S. aromaticum* EO (43.33%). At a concentration of 4%, *S. aromaticum* EO achieved a repulsion rate of 61.67%, which was comparable to that of eugenol (61.66%). At the highest concentration (6%), *S. aromaticum* EO reached 73.33% repulsion, while eugenol displayed 71.67%, indicating comparable effectiveness.

Our study shows that both *S. aromaticum* EO and eugenol exhibit significant dose-dependent repellent effects against *B. lentis* adults, aligning with findings from several studies on the repellent properties of EO and their components. For instance, Gupta et al. (2023) highlighted the effectiveness of plant-derived EOs in repelling various insect pests, underscoring the potential of natural products as viable alternatives to synthetic insecticides. Aryal et al. (2023) indicated that *S. aromaticum* EO exhibited significant repellent activity against *Sitophilus zeamais* (Coleoptera: Curculionidae). A study by Abenaim et al. (2022) reported that eugenol effectively repelled red flour beetles (*Tribolium castaneum*, Coleoptera: Tenebrionidae) at concentrations comparable to those observed in this study, further confirming its potency as a natural repellent. Additionally, da Silva et al. (2020) found that eugenol demonstrated greater efficacy against several insect species than other EOs.

### 3.6. Formulation of herbal cream

The cream, with a yellowish-white tint, has a creamy and smooth appearance, offering a soft texture that provides a refreshing sensation. It carries a fragrance with notes of clove and mastic oil. The lack of lumps or visible particles indicates effective dispersion of both the oily and aqueous phases. Analysis of the centrifuged cream shows no phase separation or signs of creaming or sedimentation, confirming the formulation’s stability, uniformity, and optimal ingredient compatibility. Microscopic examination reveals a consistent dispersion of small white droplets against a colored background, indicating the presence of oil droplets dispersed in an aqueous phase, which confirms the lipophilic/hydrophilic nature of the cream (Petsev, 2004). The droplets observed are evenly shaped, sized, and distributed, with no signs of flocculation or coalescence. The slightly acidic pH of 5.5 is perfect for skin application, helping to protect the skin barrier and maintain the stability of the active ingredients, ensuring both safety and efficacy (Salager, 2000). Lastly, the absence of microbial contamination, as shown by the enumeration of aerobic bacteria, yeasts, and molds, confirms the sterility of the formulation and the reliability of the preservative (Waqas et al., 2010).

## 4. Conclusion

The antioxidant, antibacterial, antifungal, and insecticidal activities of *Syzygium aromaticum* EO have been thoroughly evaluated and compared to eugenol, the primary component of the oil. The antioxidant assessment indicated that eugenol exhibits greater potency than the EO, with activity levels comparable to those of the standards Trolox and ascorbic acid. In lipid media, the HAT mechanism from both the OH group predominates, while in aqueous environments, eugenol tends to react through SET from its deprotonated state rather than via the HAT mechanism. Moreover, the findings regarding antibacterial and antifungal activities emphasize the superior effectiveness of the EO compared to eugenol, suggesting that eugenol is not the primary active compound responsible for these effects. Conversely, the evaluation of insecticidal activity reveals that the EO’s effectiveness is comparable to that of eugenol, indicating that eugenol is the main active component for this activity. Overall, these findings conclude that *S. aromaticum* EO possesses significant antioxidant, antibacterial, antifungal, and insecticidal properties, most of which could be attributed to its high eugenol content. Future research should focus on the exploration of the synergistic effects of minor compounds in *S. aromaticum* EO and their potential contributions to its biological activities. Additionally, further studies on in vivo applications of both EO and eugenol in various fields, such as medicine, agriculture, and food preservation, would provide valuable insights into their practical potential.

## Supplementary materials

### Formulation of herbal cream

#### 1. Macroscopic examination

The organoleptic characteristics (appearance, odor, color, and consistency) and physical stability (creaming, sedimentation, and phase separation) are examined macroscopically.

Figure S1 Macroscopic appearance of the cream.

#### 2. Microscopic examination

A drop of cream, stained with neutral red, was placed between a slide and cover slip and examined under an optical microscope (10 × 40). This examination allows for the analysis of the homogeneity of globule size distribution and detects flocculation and coalescence phenomena. The microscopic observation was performed 24 h after preparation and at the end of the study.

Figure S2 Microscopic observation of the clove essential oil-based cream.

#### 3. Centrifugation stability

The cream was centrifuged with 2 g of the product for 30 min at 3000 rpm to detect any phase separation, sedimentation, or creaming.

Figure S3 Cream after centrifugation.

#### 4. pH measurement

The pH of the cream was determined using a pH meter. Before each measurement, the pH meter was calibrated to ensure the accuracy of the results. For the measurement, approximately 0.5 g of the cream was dissolved in 50 mL of distilled water.

Figure S4 Microbiological examination results.

#### 5. Microbiological examination

The goal was to manage the bacterial and fungal contamination levels of the formulated creams, which must be sterile (**French Pharmacopoeia 11.0**), using the colony count method on agar media.

Figure S5 Final appearance of the *S. aromaticum* essential oil-based cream.

## Figures and Tables

**Table 1:** Composition of herbal cream.

Phase	Ingredient	INCI name	Properties	Quantity (%)
Aqueous phase (58.7%)	Purified water	Aqua	Main solvent for dissolving many ingredients	QSP 100
	Glycerin	Glycerin	Humectant agent (maintains hydration)	5
Oily phase (40%)	Mastic oil	*Pistacia lentiscus* fruit oil	Soothing, restorative, healing	5
	Olive oil	*Olea europaea* fruit oil	Protective, moisturizing, softening	25
	Cutina GMS	Glyceryl stearate	Emulsifying agent	8
	Stearic acid	Stearic acid	Thickening agent	2
Additives (1.3%)	Clove essential oil	*Eugenia caryophyllus* bud oil	Antioxidant, antimicrobial, anesthetic	5
	Vitamin E	Tocopherol	Antioxidant	0.2
	Cosgard	Benzyl alcohol, dehydroacetic acid	Natural preservative, broad-spectrum antibacterial and antifungal, ensures preservation of aqueous-phase preparations	0.6

**Table 2:** Phytochemical constituents contained in the EO extracted from *S. aromaticum*.

No.	Retention time (min)	Compounds	Content (%)
1	8.62	2-nonanone	0.01
2	9.80	phenylmethyl acetate	0.02
3	10.33	methyl salicylate	0.04
4	11.08	4-(1-methylethyl)-benzaldehyde	0.03
5	11.13	4-(2-propenyl)-phenol	0.06
6	11.51	(E)-cinnamaldehyde	0.01
7	11.70	(Z)-(methoxy-4-1-propenyl-benzene	0.08
8	12.71	Eugenol	79.21
9	13.04	Copaene	0.16
10	13.67	β-caryophyllene	11.60
11	14.12	Humulene	1.56
12	14.31	γ-muurolene	0.12
13	14.56	α-farmesene	0.23
14	14.69	eugenol acetate	6.27
15	14.83	1-isopropyl-4,7-dimethyl-1,2,3,5,6,8a-hexahydronaphtalene	0.27
16	15.37	1,5-dimethl-2,4-divinyl-cyclohexane	0.06
17	15.71	Caryophyllene oxide	0.21
18	16.34	11,11-dimethyl-4,8-dimethylenebicyvlo[7,2,0]undecan-3-ol	0.07

**Table 3:** Kinetic data of the reactions of eugenol and HOO^•^ radicals under physiological conditions.

Media	Mechanisms		State	ΔG^≠a^ (kcal/mol)	κ^b^	*k_app_*^c^(M^−1^s^−1^)	*f* ^d^	*k*_f_^e^ (M^−1^s^−1^)	Γ^f^ (%)	*k*_overall_ (M^−1^s^−1^)
Water	HAT	OH	**Eug**	14.3	73.1	9.00×10^3^	0.988	8.89×10^3^	0	1.16×10^7^
		CH		17.8	25.8	1.70×10^1^		1.68×10^1^	0	
	SET		**Eug** **^−^**	3.1	16.2^g^	9.70×10^8^	0.012	1.16×10^7^	100	
PE	HAT	OH	**Eug**	17.7	143.9	9.00×10^1^	-	-	-	9.00×10^1^
		CH		19.0	44.7	3.20×10^0^	-	-	-	

(a) activation energy, (b) tunneling correction, (c) apparent rate constant, (d) mole fraction, (e) *k*_f_ = *f*.*k*_app_, (f) branching ratio, and (g) the nuclear reorganization energy (λ).

**Table 4:** Antimicrobial activity of *S. aromaticum* EO and its main compound, eugenol.

Microbial strains		Diameter of inhibition zone (mm)		ANOVA	
		*S. aromaticum* EO	Eugenol	F	(p-value)
Bacterial	*B. cereus*	27.7±0.6	24.6±0.4	40.5	0.03
	*S. aureus*	31.3±0.5	21.8±0.3	649.8	<0.001
Fungal	*A. niger*	23.5±0.5	19.3±0.6	89.3	0.01
	*Trichoderma* sp.	23.2±0.3	18.5±0.5	196.0	<0.005

**Table 5:** Corrected percent mortality (mean ± SE) of *B. lentis* adults following the application of various concentrations of *S. aromaticum* EO and its main compound, eugenol, at different time intervals.

	Doses (μL/insect)	Mortality (%)		
		24 h	48 h	72 h
***S. aromaticum*** ** EO**	0.01	10.0±2.88^de^	20.0±2.88^d^	21.7±1.67^f^
	0.02	20.0±2.88^d^	30.0±2.88^d^	40.0±0.67^e^
	0.04	60.0±2.88^b^	70.0±1.66b^c^	80.0±2.88^bc^
	0.06	90.0±2.88^a^	96.66±3.33^a^	100^a^
***Eugenol***	0.01	36.66±3.33^c^	56.67±3.33^c^	60.0±0.0^d^
	0.02	56.78±3.33^b^	66.67±3.33^c^	73.33±3.33^c^
	0.04	66.67±3.33^b^	80.0±0.0^b^	83.33±3.33^b^
	0.06	93.33±3.33^a^	100^a^	100^a^
***Control (acetone)***	0.01	0	0	0
	0.02	0	0	0
	0.04	0	0	0
	0.06	0	0	0

**Table 6:** Lethal concentrations for the contact toxicity of *S. aromaticum* EO and its main compound, eugenol against *B. lentis* at different exposure times.

Treatment	Exposure time (h)	LC_50_ a(μL/insect)	LC_90_ a(μL/insect)	Slope ± SEM b	Chi-square (χ2)	df
EO	24	0.031 (0.021–0.041)	0.075 (0.049–0.288)	5.74±1.73	0.91	2
	48	0.024 (0.015–0.034)	0.060 (0.039–0.219)	5.5±1.60	2.55	2
	72	0.021 (0.013–0.030)	0.049 (0.033–0.157)	5.78±1.67	1.37	2
Eugenol	24	0.016 (0.003–0.028)	0.079 (0.041–7.14)	1.86±0.75	1.12	2
	48	0.01 (0.00003–0.017)	0.047 (0.026–13.31)	1.86±0.82	1.53	2
	72	0.008 (0.000007–0.016)	0.041 (0.023–42.42)	1.84±0.85	1.19	2

LC: lethal concentration;

a Values in the bracket represent lower and upper confidence limit;

b SEM: Standard error of the mean.

**Table 7:** Repellent activity (%) of *S. aromaticum* EO and its main compound, eugenol against *B. lentis* at different concentrations.

Concentration	Repelled adults (%) after 2 h of exposure	
	*S. aromaticum* EO	Eugenol
1%	25.0±2.88^d^	26.70±3.33^cd^
2%	43.33±3.33^c^	51.67±1.67^bc^
4%	61.67±1.67^ab^	61.66±1.67^ab^
6%	73.33±3.33^a^	71.67±1.67^a^

Means within the same column followed by same letter are not significantly different (p < 0.05).
