# Supplementary figures and images for: Evaluating the multifaceted bioactivity of Syzygium aromaticum essential oil: the central role of eugenol
Source: Turk J Biol. 2025 Jan 13;49(1):102–17. doi: 10.55730/1300-0152.2728 (PMC11913364; doi:10.55730/1300-0152.2728)

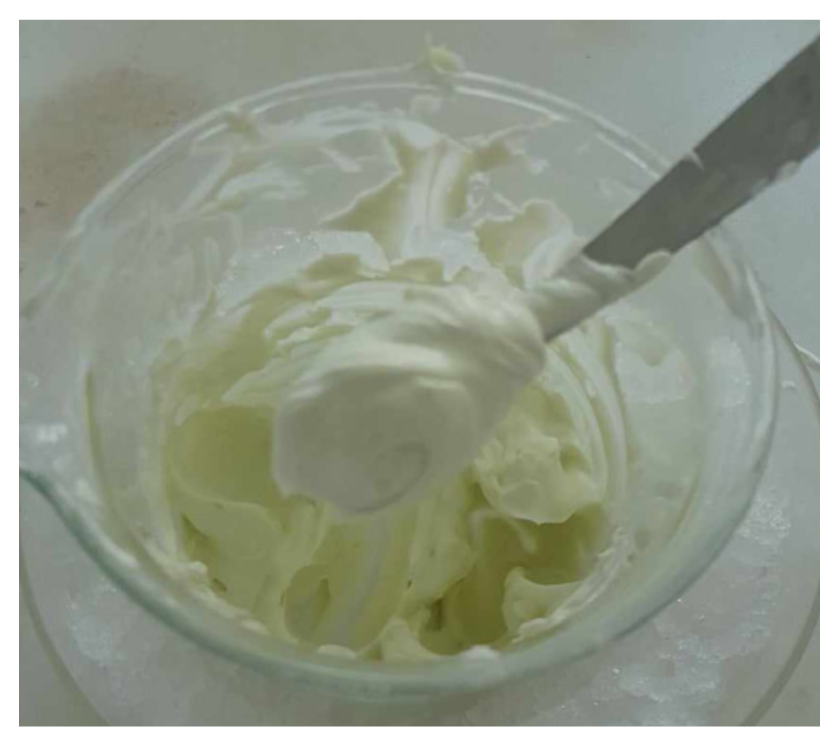

Supplement: Figure S1 — Macroscopic appearance of the cream. [file tjb-49-01-102s1.tif]

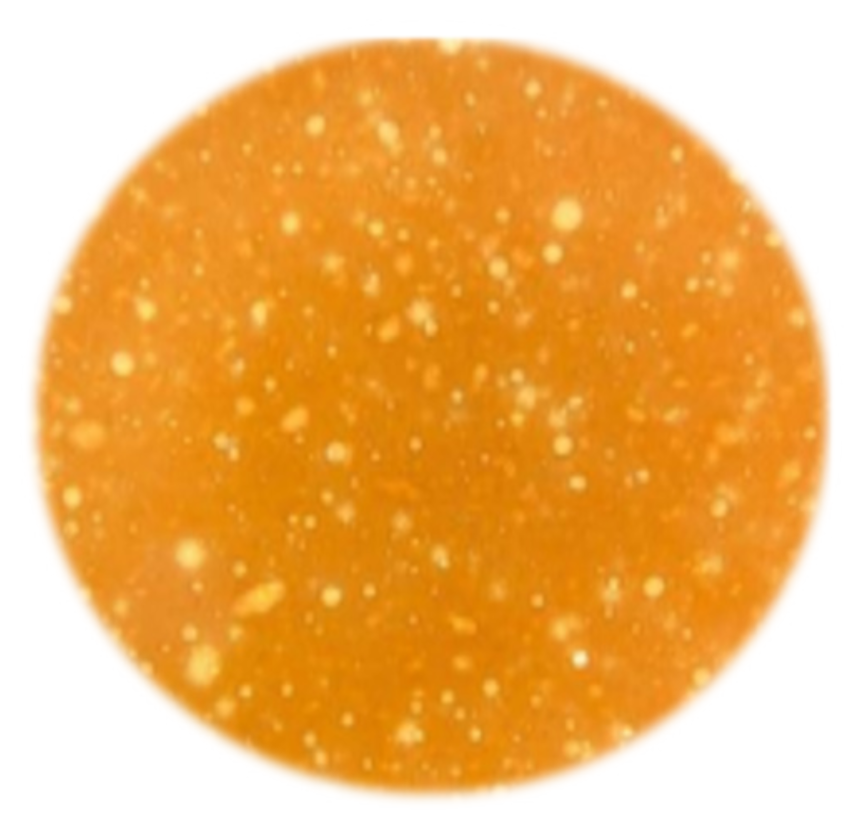

Supplement: Figure S2 — Microscopic observation of the clove essential oil-based cream. [file tjb-49-01-102s2.tif]

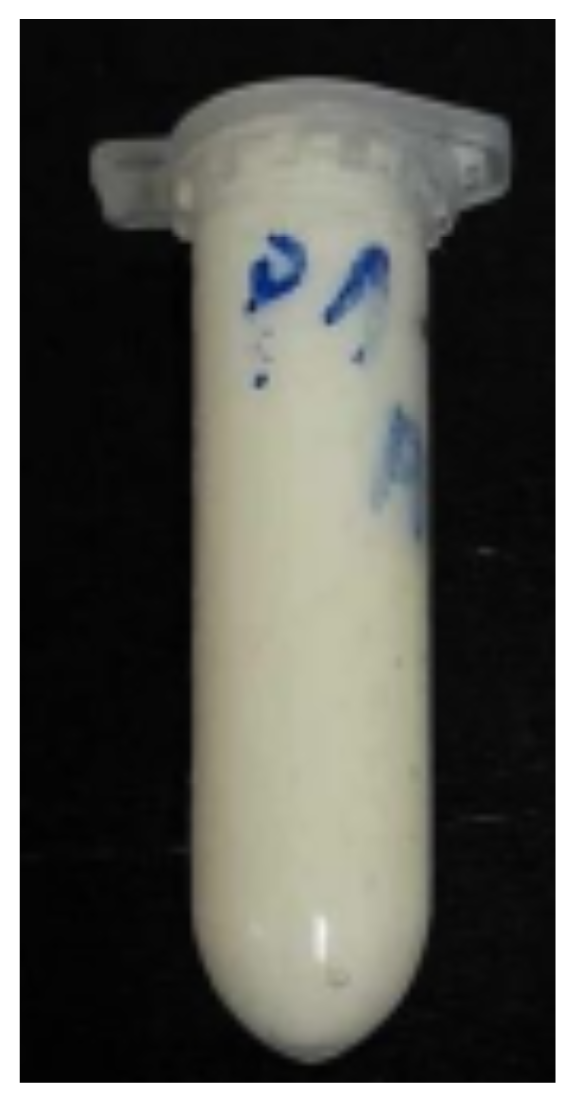

Supplement: Figure S3 — Cream after centrifugation. [file tjb-49-01-102s3.tif]

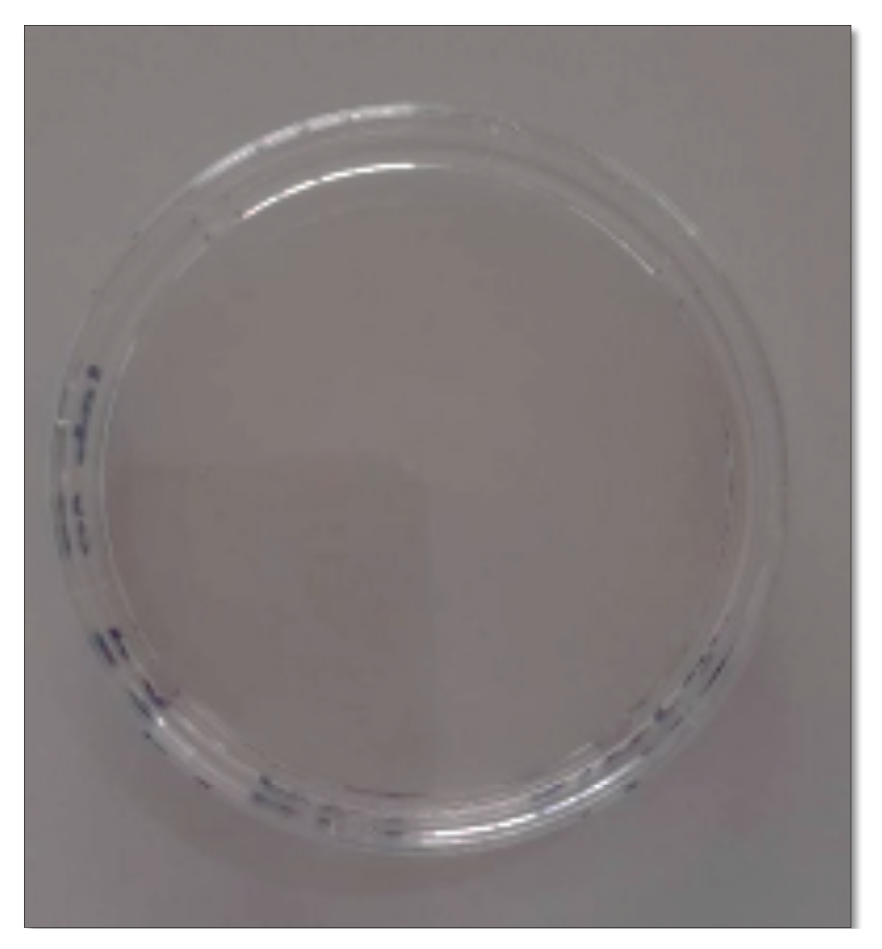

Supplement: Figure S4 — Microbiological examination results. [file tjb-49-01-102s4.tif]

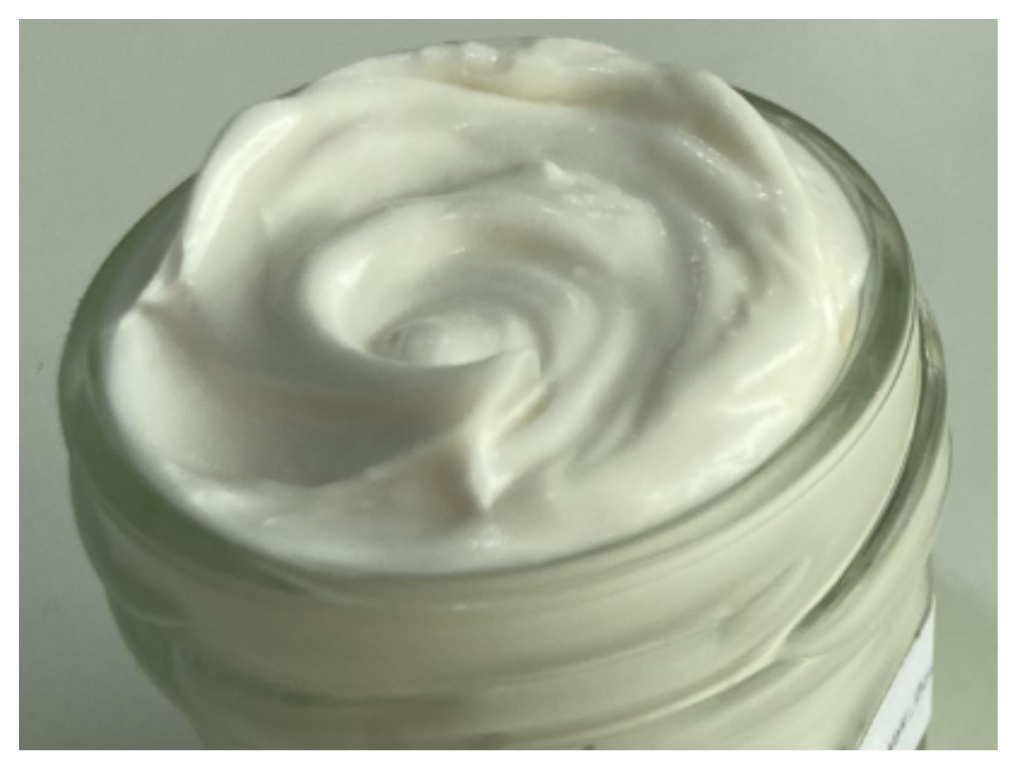

Supplement: Figure S5 — Final appearance of the S. aromaticum essential oil-based cream. [file tjb-49-01-102s5.tif]
